# Subunit-specific mutational analysis of residue N348 in HIV-1 reverse transcriptase

**DOI:** 10.1186/1742-4690-8-69

**Published:** 2011-08-22

**Authors:** Jessica Radzio, Nicolas Sluis-Cremer

**Affiliations:** 1 University of Pittsburgh School of Medicine, Department of Medicine, Division of Infectious Diseases, Pittsburgh, PA 15261, USA; 2 University of Pittsburgh Graduate School of Public Health, Department of Infectious Diseases and Microbiology, Pittsburgh, PA 15261, USA

## Abstract

**Background:**

N348I in HIV-1 reverse transcriptase (RT) confers resistance to zidovudine (AZT) and nevirapine. Biochemical studies demonstrated that N348I indirectly increases AZT resistance by decreasing the frequency of secondary ribonuclease H (RNase H) cleavages that reduce the RNA/DNA duplex length of the template/primer (T/P) and diminish the efficiency of AZT-monophosphate (MP) excision. By contrast, there is some discrepancy in the literature in regard to the mechanisms associated with nevirapine resistance: one study suggested that it is due to decreased inhibitor binding while others suggest that it may be related to the decreased RNase H cleavage phenotype. From a structural perspective, N348 in both subunits of RT resides distal to the enzyme's active sites, to the T/P binding tract and to the nevirapine-binding pocket. As such, the structural mechanisms associated with the resistance phenotypes are not known.

**Results:**

Using a novel modelled structure of RT in complex with an RNA/DNA T/P, we identified a putative interaction between the β14-β15 loop in the p51 subunit of RT and the RNA template. Substitution of the asparagine at codon 348 in the p51 subunit with either isoleucine or leucine abrogated the observed protein-RNA interaction, thus, providing a possible explanation for the decreased RNase H phenotype. By contrast, alanine or glutamine substitutions exerted no effect. To validate this model, we introduced the N348I, N348L, N348A and N348Q mutations into RT and purified enzymes that contained subunit-specific mutations. N348I and N348L significantly decreased the frequency of secondary RNase H cleavages and increased the enzyme's ability to excise AZT-MP. As predicted by the modelling, this phenotype was due to the mutation in the p51 subunit of RT. By contrast, the N348A and N348Q RTs exhibited RNase H cleavage profiles and AZT-MP excision activities similar to the wild-type enzyme. All N348 mutant RTs exhibited decreased nevirapine susceptibility, although the N348I and N348L mutations conferred higher fold resistance values compared to N348A and N348Q. Nevirapine resistance was also largely due to the mutation present in the p51 subunit of RT.

**Conclusions:**

This study demonstrates that N348I-mediated AZT and nevirapine resistance is due to the mutation in the p51 subunit of RT.

## Background

HIV-1 reverse transcriptase (RT) is a key target for antiretroviral drug development. To date, 12 RT inhibitors (RTIs) have been approved for the treatment of HIV-1 infection that can be classified into 2 distinct therapeutic groups [1]. These include: (i) the nucleoside/nucleotide RT inhibitors (NRTI) that bind to the DNA polymerase active site of the enzyme and act as competitive inhibitors of DNA polymerization [2]; and (ii) the nonnucleoside inhibitors (NNRTI) that bind to a non-active site pocket in HIV-1 RT (termed the NNRTI-binding pocket) and act as allosteric inhibitors of DNA polymerization [3]. Although combination therapies that contain two or more RTI have profoundly reduced morbidity and mortality from HIV-1 infection, their long-term efficacy is limited by the selection of drug-resistant variants of HIV-1.

**Figure 1:**
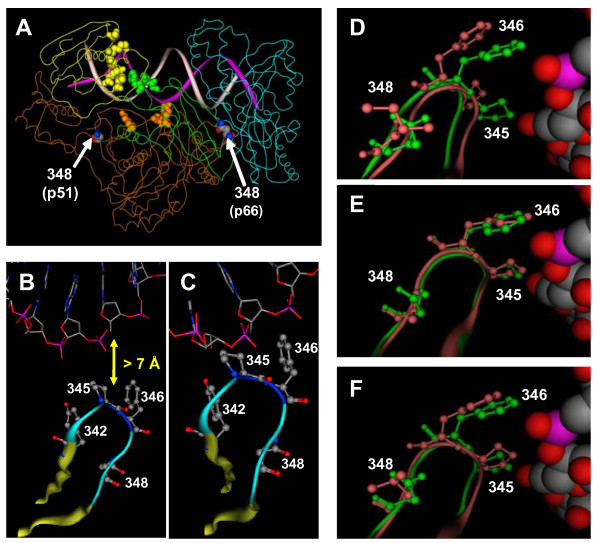
**Interaction between residue N348 in the p51 subunit of HIV-1 RT and the RNA template**. A) Crystal structure of HIV-1 RT in complex with a polypurine tract RNA/DNA T/P (pdb accession code 1HYS). The p66 DNA polymerase, connection and RNase H domains are colored cyan, green and yellow, respectively. The p51 subunit is colored orange. The DNA and RNA strands are colored white and purple, respectively. Residues in the connection and RNase H domain that form part of the nucleic acid binding tract are shown in spacefill (and colored according to domain color). B) Location of the β14-β15 loop in the p51 subunit of HIV-1 RT in complex with a PPT RNA/DNA hybrid. Residues 345 and 346 reside > 7Å from the RNA strand. C) Location of the β14-β15 in the p51 subunit of HIV-1 RT in complex with an RNA/DNA duplex that extends into the RNase H active site. Residues 345 and 346 all directly contact the RNA template. The co-ordinates for the model were kindly provided by Dr M. Nowotny. D, E, F) Impact of the N348I (D), N348A (E) and N348L (F) mutations on the β14-β15 loop in the p51 subunit of HIV-1 RT in complex with an RNA/DNA duplex that extends into the RNase H active site. The WT and mutant structures are colored green and pink, respectively. The co-ordinates for this structure were kindly provided by Dr M. Nowotny (NIDDK, NIH). Mutations were introduced into this structure using MOE. Charges were calculated using the Gasteiger method, and iterative minimizations were carried out using the AMBER 99 force field until the energy difference between iterations was less than 0.0001 kcal/mol per Å.

HIV-1 RT is a heterodimer composed of a 66 kDa subunit (p66), and a p66-derived 51 kDa subunit (p51) [4]. The catalytically active p66 subunit of RT consists of DNA polymerase, connection and ribonuclease H (RNase H) domains. Most of the RTI resistance mutations identified to date map to the DNA polymerase domain of RT. However, a growing body of evidence has emerged that implicates mutations outside of the polymerase domain of RT in RTI resistance [5]. In this regard, the N348I mutation in the connection domain of HIV-1 RT has received significant attention in the last 4 years. This mutation can be selected relatively early during virologic failure and confers resistance to both zidovudine (AZT) and nevirapine [6]. Furthermore, N348I can compensate for the antagonism of thymidine analog mutations (TAMs) by the L74V, Y181C or M184V mutations [7]. Previous biochemical studies demonstrated that N348I in HIV-1 RT indirectly increases AZT resistance by decreasing the frequency of secondary ribonuclease H (RNase H) cleavages that significantly reduce the RNA/DNA duplex length of the template/primer (T/P) and diminish the efficiency of AZT-monophosphate (MP) excision [6,8]. By contrast, there is some discrepancy in the literature in regard to the mechanisms associated with nevirapine resistance: one study has suggested it is due to decreased inhibitor binding [9], while other studies suggest that it may also be due to the decreased RNase H cleavage phenotype of the N348I HIV-1 RT [10,11]. Interestingly, in the available crystal structures of HIV-1 RT, residue N348 in both subunits of the enzyme is located distal to the DNA polymerase and RNase H active sites, to the T/P substrate, to residues that comprise the nucleic acid binding tract and to the NNRTI-binding pocket [Figure 1A, B]. Therefore, it is not evident how N348I in HIV-1 RT impacts the RNase H cleavage of the enzyme or decreases drug susceptibility. In this study, we used a combination of molecular modelling and biochemical analyses to address this question.

## Results and Discussion

### Molecular models of wild type (WT) and N348 mutant HIV-1 RT in complex with an RNA/DNA T/P

In the crystal structure of HIV-1 RT in complex with a polypurine tract RNA/DNA hybrid [12], residue N348 in both subunits is not proximal to the enzyme's active sites, to the RNA/DNA T/P substrate, to residues that comprise the nucleic acid binding tract and to the NNRTI-binding pocket [Figure 1A, B]. Accordingly, the mechanisms by which N348I decreases RT RNase H activity and drug susceptibility cannot be inferred from this structure. It should, however, be noted that although the RNA/DNA duplex extends into the RNase H domain of RT in this structure, it misses the active site by ~ 4 Å. Recently, a crystal structure of the human RNase H1 was solved in complex with an RNA/DNA substrate which extends directly into the enzyme's active site [13]. Because of the similarity between the human RNase H1 and the RNase H domains of HIV-1 RT, the authors were able to model an RNA/DNA duplex into HIV-1 RT that extends from the RNase H active site of the enzyme. It should be noted that due to the orientation and conformation of the bound T/P in this model, HIV-1 RT cannot simultaneously carry-out DNA polymerization and RNase H cleavage. Accordingly, it was proposed that the RNA/DNA T/P substrate would need to toggle between both active sites [13]. A recent study by Beilhartz *et al.*, however, refutes this hypothesis [14]. Nevertheless, in this model, residues Y342, P345 and F346 from the β14-β15 loop of the p51 subunit of HIV-1 RT directly interact with the RNA template backbone [Figure 1C]. The C^β ^atom of N348 forms a network of interactions with the C^β ^atom and backbone atoms of Y342. [N348 in p66 remains distal to the RNA/DNA substrate in this model (data not shown)]. When the N348I mutation is introduced into the p51 subunit in this structure by molecular modelling (Figure 1D), the position of the β14-β15 loop is shifted such that P345 and F346 no longer contact the RNA template. The repositioning of this loop in the N348I RT is likely due to the bulky side-chain of isoleucine disrupting the network of interactions between this residue and Y342. Similarly, the introduction of leucine (Figure 1F) or glutamic acid (data not shown) at residue 348 in the p51 subunit of RT resulted in a shift of the β14-β15 loop away from the RNA template. By contrast, introduction of alanine (Figure 1E) or glutamine (data not shown) had little impact on the position of this loop. Both these substitutions retain the critical network of interactions between residue 348 and 342. Interestingly, the introduction of an arginine residue appeared to enhance the interactions of the P345 and F346 with the RNA template (data not shown). Taken together, these modelling studies suggest that the N348I mutation in the context of the p51 subunit of HIV-1 RT may decrease the enzyme's RNase H activity via an altered interaction with the RNA template. Importantly, these modelling analyses provided a testable hypothesis.

### Subunit-specific mutational analysis of residue N348 in HIV-1 RT

**Figure 2:**
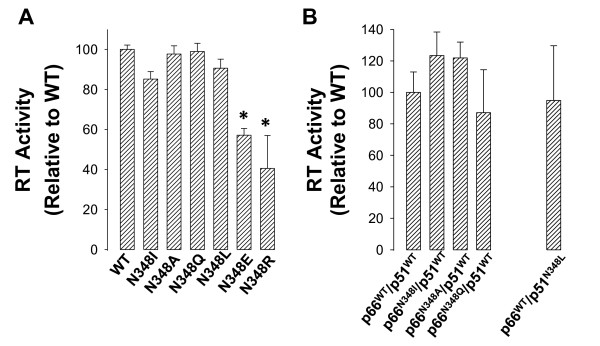
**DNA polymerase activity of recombinant purified HIV-1 RT that contained mutations at residue N348 in both subunits (A), or in only one subunit (B), of the enzyme**. The DNA polymerase activity was assessed as described in the Methods. Data are reported as an average ± standard deviation of at least 3 separate experiments. An asterisk indicates *P *< 0.01 compared with WT (Student's *t*-test).

As described above, our modelling data suggested that the RNase H activity of HIV-1 RT could be modulated by mutations at residue N348 in the p51 subunit of the enzyme. Accordingly, we generated by site directed mutagenesis six HIV-1 RT constructs that contained the N348I, N348A, N348Q, N348L, N348E or N348R mutations. Initially, enzymes that harbored the mutations in both subunits were purified to homogeneity and assessed for RNA-dependent DNA polymerase activity (Figure 2A). The DNA polymerase activities of N348A, N348I, N348L and N348Q were found to be similar to that of the WT enzyme. By contrast, the polymerase activities of the N348E and N348R RTs were decreased significantly compared to the WT enzyme, and accordingly they were excluded from subsequent analyses. Next, we over-expressed and purified RTs that contained subunit-specific mutations using the pDUET expression vector (see Methods). In this regard, we successfully purified the p66^N348I^/p51^WT^, p66^N348A^/p51^WT^, p66^N348Q^/p51^WT ^and p66^WT^/p51^N348L ^enzymes. Importantly, the DNA polymerase activities of these purified enzymes were similar to that of the WT enzyme purified under the same conditions (Figure 2B). Unfortunately, we were unable to purify several other subunit-specific combinations due to low expression levels, protein insolubility, or inability of the p66 and p51 subunits to form functional RT heterodimers. To determine whether an alternate purification strategy would be viable, we also expressed the p66 and p51 subunits separately (see Methods). The bacterial lysates were then mixed and HIV-1 RT purified using a dual tag strategy that involved nickel- and FLAG-affinity chromatography. This alternate approach, however, was also unsuccessful. Previously Schuckman *et al. *described the purification of p66^WT^/p51^N348I ^HIV-1 RT [9]. In this regard, it is important to note that their approach involved nickel affinity and mono Q anion exchange chromatography, and not the dual tag affinity strategy used in our study. Because the p66 subunit of RT can be cleaved to p51 by bacterial proteases, one cannot exclude the possibility that the purified enzymes prepared by Schuckman and co-workers were not contaminated by p66^N348I^/p51^N348I ^HIV-1 RT.

### ATP mediated excision of AZT-MP from a chain-terminated T/P by WT and N348 mutant HIV-1 RT

**Figure 3:**
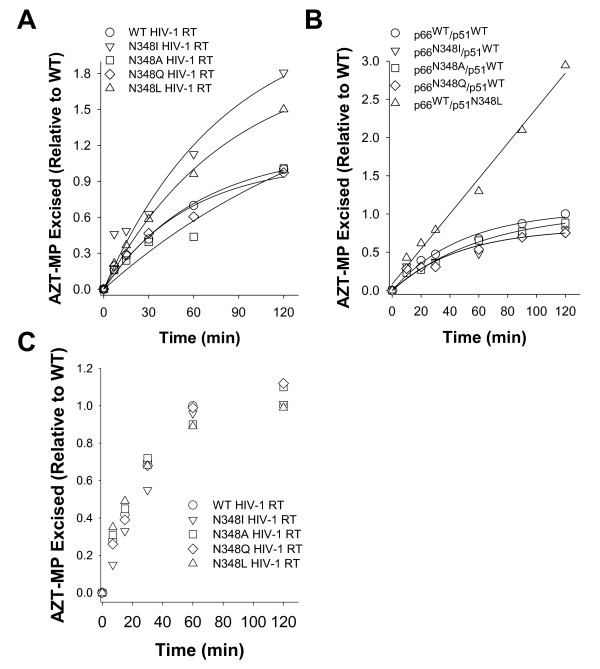
**AZT-MP excision activity of HIV-1 RT containing mutations at residue 348**. A) Time course of ATP-mediated AZT-MP excision reactions carried out by HIV-1 RT containing mutations at residue N348 in both subunits of the enzyme on an RNA/DNA T/P. Data are the mean ± standard deviation from at least three independent experiments. B) Time course of ATP-mediated AZT-MP excision reactions carried out by HIV-1 RT containing mutations at residue N348 in only one subunit of the enzyme on an RNA/DNA T/P. Data are the mean ± standard deviation from at least three independent experiments. C) Time course of ATP-mediated AZT-MP excision reactions carried out by HIV-1 RT containing mutations at residue N348 in both subunits of the enzyme on a DNA/DNA T/P. Data are the average from at least two independent experiments.

**Figure 4:**
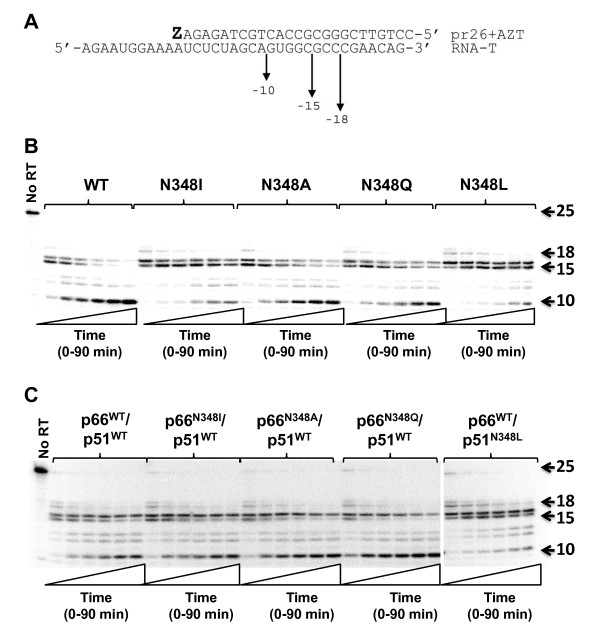
**RNase H cleavage activity of recombinant purified HIV-1 RT that contained mutations at residue N348**. A) Schematic illustrating the location of the RNase H cleavage sites in the RNA template. B) Autoradiogram of the RNase H cleavage patterns of HIV-1 RTs containing mutations at residue 348 in both subunits of the enzyme. Experiments were carried out as described in the Methods. The reaction times were 1, 3, 5, 10, 20, 30, 45, 60 and 90 min. C) Autoradiogram of the RNase H cleavage patterns of HIV-1 RTs containing mutations at residue 348 in either the p66 or p51 subunits of the enzyme. Experiments were carried out as described in the Methods. The reaction times were 1, 3, 5, 10, 20, 30, 45, 60 and 90 min.

TAMs in HIV-1 RT confer AZT resistance by enabling the enzyme to excise the chain-terminating AZT-MP moiety from the 3'-end of the DNA primer using ATP as a phosphate donor [15]. Previous biochemical studies demonstrated that N348I in HIV-1 RT indirectly increases AZT resistance by decreasing the frequency of secondary RNase H cleavages that significantly reduce the RNA/DNA duplex length of the T/P and diminish the efficiency of AZT-MP excision [6,8]. As such, we first assessed the AZT-MP excision activity of the WT and N348 mutant enzymes on a well-defined RNA/DNA T/P substrate that is routinely used in our laboratory [6,7,16,17]. When the mutation at residue 348 was present in both the p66 and the p51 subunits of RT, only the N→I and N→L substitutions conferred an enhanced ability to excise AZT-MP compared to the WT enzyme (Figure 3A). However, when N348I was present only in the p66 subunit, the mutant enzyme exhibited AZT-MP excision activity that was similar to the WT RT (Figure 3B). By contrast, when N348L was present in the p51 subunit only, the mutant enzyme exhibited robust AZT-MP excision activity (Figure 3B). As predicted by the molecular modelling studies, the N348A and N348Q mutations had minimal impact on the ATP-mediated excision activity of the enzyme (Figures 3A, B). As expected, the mutant RTs exhibited ATP-mediated excision activities on a DNA/DNA T/P substrate that were comparable to the WT enzyme (Figure 3C). We previously delineated the relationship between AZT-MP excision efficiency and RNase H activity on the RNA/DNA T/P substrate used in these experiments [16,17]. These studies showed that the primary polymerase-dependent RNase H cleavage of RT does not impact the enzyme's AZT-MP excision efficiency, but polymerase-independent RNase H cleavages that reduce the RNA/DNA duplex length to less than 12 nucleotides abolish AZT-MP excision activity. In light of these data, we next evaluated the RNase H activity of WT and N348 mutant RT that occurred during the ATP-mediated excision reactions described in Figure 3. As reported previously [6,8], N348I significantly reduced the frequency of a polymerase-independent cleavage event that decreases the RNA/DNA duplex to 10 nucleotides (Figure 4B). Consistent with the observed increase in AZT-MP excision activity, the N348L mutation also significantly decreased the frequency of this polymerase-independent cleavage event. By contrast, the N348A and N348Q enzymes retained near WT-like RNase H cleavage activities. Interestingly, when the N348I mutation was present only in the p66 subunit, the observed RNase H cleavage pattern was similar to that of the WT enzyme (Figure 4C). By contrast, when N348L was present only in the p51 subunit, the mutant enzyme showed an RNase H phenotype similar to the enzyme that contained the N348L mutation in both subunits (Figure 4B, C). Taken together, these data show that the N348I or N348L mutations in the p51 subunit of HIV-1 RT are responsible for the observed decreased RNase H cleavage and increased AZT-MP excision phenotypes. This finding is consistent with that of Schuckmann *et al. *who also reported that the N348I mutant in the p51 subunit conferred the decreased RNase H/increased AZT-MP excision phenotype [9].

### Susceptibility of WT and N348 mutant HIV-1 RT to nevirapine

Next, we determined the susceptibility of the WT and N348 mutant HIV-1 RT to nevirapine (Table 1). Our results show that the N348A, N348I, N348Q and N348L mutations confer nevirapine resistance when present in both the p66 and p51 subunits of HIV-1 RT. However, it should be noted that HIV-1 RTs that contained the N348I and N348L mutations showed fold-changes in nevirapine resistance (8.6- and 11.4-fold, respectively) that were greater than those calculated for the N348A (3.4-fold) or N348Q (2.4-fold) HIV-1 RTs. Interestingly, when the N348A, N348Q or N348I mutations were present in the p66 subunit only, RT susceptibility to nevirapine was similar to that of the WT enzyme (Table 1). By contrast, when N348L was present in p51 only, HIV-1 RT exhibited 5.7-fold nevirapine resistance. Taken together, these data strongly suggest that nevirapine resistance, like AZT resistance, is also due to the mutation in the p51 subunit of RT. It should be noted, however, that the fold-change in nevirapine resistance was lower for the p66_WT_/p51^N348L ^RT (5.7-fold) compared to p66^N348L^/p51^N348L ^RT (11.4-fold). As such, the mutation in the p66 subunit may augment nevirapine resistance. Nikolenko *et al. *proposed that the decrease in RNase H activity due to mutations in the connection or RNase H domains of HIV-1 RT preserves the RNA template and provides more time for NNRTIs to dissociate from the RT, resulting in the resumption of DNA synthesis and enhanced NNRTI resistance [11]. Of note, our data show that the two mutations that have a significant impact on the RNase H phenotype (i.e. N348I and N348L) also confer the highest levels of nevirapine resistance, thus providing additional support for a link between RNase H cleavage and NNRTI resistance. However, the N348A and N348Q mutations also yielded nevirapine resistance. Since these mutations do not significantly impact the RNase H phenotype, one must conclude that they confer resistance via an RNase H independent phenotype. Interestingly, previous studies have demonstrated that N348I confers nevirapine resistance on both RNA/DNA and DNA/DNA T/P substrates [9,10], suggesting that factors in addition to RNase H cleavage impact nevirapine binding. Recently, Schuckmann *et al. *reported that the N348I mutation in either subunit caused nevirapine resistance [9]. Specifically, they showed that the mutation in p66 alone caused nevirapine resistance without significantly affecting RNase H activity, whereas the mutation in p51 caused nevirapine resistance and impaired RNase H activity. The nevirapine fold-resistance values (~ 2-3 fold) determined by Schuckmann *et al. *were significantly less than the values determined in our study. Furthermore, they reported approximately ~ 2-2.7 -fold resistance when the N348I mutation was present in both subunits; ~ 1.9-2-fold when present in p66 only; and 2.7-3.1-fold when present in p51 only (see Table three in [9]). If nevirapine resistance was due to the mutation in both subunits, one would expect a higher fold-resistance for the p66^N348I^/p51^N348I ^RT compared to the p66^N348I^/p51^WT ^or p66^WT^/p51^N348I ^enzymes. As mentioned previously, the purification strategy used by Schuckmann *et al. *did not employ an affinity chromatography step that was specific for each subunit. As such, their enzyme preparations may have been contaminated by p66^N348I^/p51^N348I ^RT generated by bacterial proteases, thus complicating data analysis.

**Table 1:** Susceptibility of WT and N348 mutant HIV-1 to nevirapine

Enzyme	IC_50 _(μM)	Fold Resistance
p66^WT^/p51^WT^	0.52 ± 0.04	-

p66^N348A^/p51^N348A^	1.77 ± 0.50	3.4

p66^N348A^/p51^WT^	0.62 ± 0.07	1.2

p66^N348I^/p51^N348I^	4.60 ± 0.45	8.6

p66^N348I^/p51^WT^	0.50 ± 0.11	0.97

p66^N348L^/p51^N348L^	5.93 ± 0.54	11.4

p66^WT^/p51^N348L^	2.96 ± 0.10	5.7

p66^N348Q^/p51^N348Q^	1.25 ± 0.13	2.4

p66^N348Q^/p51^WT^	0.36 ± 0.05	0.7

## Conclusions

This study demonstrates that N348I-mediated AZT and nevirapine resistance is likely due to the mutation in the p51 subunit of RT. It should be noted, however, that the interpretation of our data is limited by our inability to purify and characterize p66^WT^/p51^N348I ^HIV-1 RT. The molecular modelling suggests that the N348I mutation abrogates an interaction between the β14-β15 loop in the p51 subunit of RT and the RNA template, which may explain the observed decrease in the secondary or polymerase independent RNase H cleavages. Nevirapine resistance appears complex and may involve both RNase H-dependent and -independent mechanisms.

## Methods

### Molecular modelling

The co-ordinates for the molecular model of HIV-1 RT in complex with an RNA/DNA duplex that extends into the RNase H active site of the enzyme [13] were kindly provided by Dr. Marcin Nowotny. To generate the models, we first selected the N348 residue in the p51 subunit and selected all amino acid residues within a 20 Å radius. These residues were then subjected to energy minimization using the Molecular Operating Environment (Chemical Computing Group, Montreal, Quebec, Canada). The rest of RT and the RNA/DNA T/P substrate were frozen and were not subjected to energy minimization. Charges were calculated using the Gasteiger method, and iterative minimizations were carried out using the AMBER 99 forcefield until the energy difference between iterations was less than 0.0001 kcal/mol per Å. These initial minimization experiments had minimal effect on the overall structure of the RT. Next, we introduced the N348I/A/Q/E/R/L mutations into the p51 subunit of HIV-1 RT, and carried out energy minimizations as described above. Modeled RT structures were visualized using MOE or the UCSF Chimera package from the Resource for Biocomputing, Visualization, and Informatics at the University of California, San Francisco (supported by NIH P41 RR001081).

### Reagents

AZT- TP was purchased from Sierra Bioresearch (Tuscon, AZ). Nevirapine was obtained from the NIH AIDS Research and Reference Reagent Program. ATP, dNTPs, and ddNTPs were purchased from GE Healthcare (Piscataway, NJ), and [γ-^32^P]ATP was acquired from PerkinElmer Life Sciences (Boston, MA). RNA and DNA oligonucleotides were synthesized by Integrated DNA Technologies (Coralville, IA).

### Site-directed mutagenesis and protein expression

The N348A, N348E, N348L, N348Q and N348R mutations were introduced into the wild-type (WT) p6HRT-Prot prokaryotic expression vector [18] by site-directed mutagenesis using the QuikChange mutagenesis kit (Stratagene La Jolla, CA). Full-length sequencing of mutant RTs was performed to confirm the presence of the desired mutations and to exclude adventitious mutations introduced during mutagenesis. The mutant HIV-1 RTs were purified as described previously [18]. For subunit selective mutagenesis, the p66 and p51 RT genes were cloned into the pET-DUET vector (Novagen-EMD Biosciences Inc., San Diego, California). The p66 subunit was expressed as an N-terminal hexahistidine fusion protein whereas p51 was expressed as an N-terminal FLAG fusion protein. The p66 and p51 subunits of RT were also cloned into the pBAD/His B (Invitrogen) and pT7-FLAG (Sigma) expression vectors to generate His-p66 and FLAG-p51, respectively. Enzymes were expressed and purified as described previously using a double-tag strategy [19]. The protein concentration of the purified enzymes was determined spectrophotometrically at 280 nm using an extinction coefficient (ε_280_) of 260450 M^-1 ^cm^-1^, and by Bradford protein assays (Sigma-Aldrich, St. Louis, MO).

### AZT-MP excision assays

A 26-nucleotide DNA primer (**pr26**; 5'-CCTGTTCGGGCGCCACTGCTAGAGAT-3') was 5'-radiolabeled with [γ-^32^P]ATP and chain-terminated with AZT-MP to generate P_AZT _as reported previously [6,7,16,17]. P_AZT _was then annealed to a 35-nucleotide RNA template (T_RNA_: 5'-AGAAUGGAAAAUCUCUAGCAGUGGCGCCCG AACAG-3'). ATP-mediated AZT-MP excision assays were carried out by first incubating 20 nM T_RNA_/P_AZT _with 3 mM ATP, 10 mM MgCl_2_, 1 μM dTTP and 10 μM ddCTP in a buffer containing 50 mM Tris-HCl (pH 7.5) and 50 mM KCl. Reactions were initiated by the addition of 200 nM WT or mutant RT. Aliquots were removed at defined times, quenched with sample loading buffer (98% deionized formamide, 1 mg/ml each of bromophenol blue and xylene cyanol), denatured at 95°C for 8 min, and then product was resolved from substrate by denaturing polyacylamide gel electrophoresis and analyzed, as reported previously [6,7,16,17].

### Assay for RT RNase H activity

WT and mutant RT RNase H activity was evaluated using the same AZT-MP chain-terminated RNA/DNA T/P substrate described above, except the 5'-end of the RNA was ^32^P-end-labelled. Assays were carried out using 20 nM T_RNA_/P_AZT_, 3 mM ATP and 10 mM MgCl_2 _in a buffer containing 50 mM Tris-HCl (pH 7.5) and 50 mM KCl. Reactions were initiated by the addition of 200 nM WT or mutant HIV-1 RT. Aliquots were removed, quenched at varying times, and analyzed as described above.

### Inhibition of WT and N348 mutant HIV-1 RT by nevirapine

Fixed time point assays using a heteropolymeric T/P substrate were used to determine HIV-1 RT DNA polymerase activity, as reported previously [20]. The sequence of the DNA primer and RNA template were 5'-TCGGGCGCCACTGCTAGAGA-3' and 5'-UCAGACCCUUUUAGUCAGAAUGGAAAGUCUCUAGCAGUGGCGCCCGAACAGGGACA-3', respectively. The primer was synthesized with a biotin label on their 5'-terminus. DNA polymerase reactions using heteropolymeric T/P (600 nM) were carried out in 50 mM Tris-HCl pH 7.5 (37°C), 50 mM KCl, 10 mM MgClB_2B _containing 600 nM T/P, 10 μM of each [P^3P^H]dNTP, and variable concentrations of nevirapine (0-20 μM). Reactions were initiated by the addition of 25 nM of RT, incubated for 20 min at 37°C and then quenched with 0.5 M EDTA. Streptavidin Scintillation Proximity Assay beads (GE Healthcare, Piscataway, NJ) were then added to each reaction, and the extent of radionucleotide incorporation was determined by scintillation spectrometry using a 1450 Microbeta Liquid Scintillation Counter (Perkin Elmer, Waltham, MA).

## Competing interests

The authors declare that they have no competing interests.

## Authors' contributions

Conceived and designed the experiments: JR and NSC. Performed the experiments: JR. Analyzed the data: JR and NSC. Wrote the paper: NSC. All authors read and approved the final manuscript.
